# Supplementary material for: Drug-Associated Parosmia: New Perspectives from the WHO Safety Database
Source: J Clin Med. 2022 Aug 9;11(16):4641. doi: 10.3390/jcm11164641 (PMC9409668; doi:10.3390/jcm11164641)
Supplement: Supplementary file 1 [file jcm-11-04641-s001.zip › jcm-1853796-supplementary.pdf]

Table S1. Reported suspected drugs in patients with parosmia

Drugs	Number (%)
**COVID-19 vaccine**	**3,796 (27.1)**
Tozinameran	2,103 (55.4)
Elasomeran	745 (19.6)
AZD1222	724 (19.1)
JNJ 78436735	191 (5.0)
Others	33 (0.9)
Clarithromycin	288 (2.1)
Varenicline	245 (1.7)
Fluticasone	223 (1.6)
Azithromycin	152 (1.1)
Beclometasone	145 (1.1)
Adalimumab	131 (0.9)
Roxithromycin	125 (0.9)
Ribavirin	121 (0.9)
Terbinafine	121 (0.9)
Ciprofloxacin	119 (0.8)
Ipratropium	113 (0.8)
Mometasone	106 (0.8)
HPV vaccine	106 (0.8)
Etanercept	101 (0.7)
Levofloxacin	100 (0.7)
Oxymetazoline	99 (0.7)
Moxifloxacin	95 (0.7)
Teriparatide	95 (0.7)
Ofloxacin	91 (0.6)
Flunisolide	83 (0.6)
Duloxetine	82 (0.6)
Lenalidomide	82 (0.6)
Bupropion	81 (0.6)
Salbutamol	78 (0.6)
Doxycycline	78 (0.6)
Influenza vaccine	75 (0.5)
Exenatide	71 (0.5)
Omeprazole	70 (0.5)
Levonorgestrel	70 (0.5)
Paroxetine	66 (0.5)
Metronidazole	60 (0.4)
Levothyroxine	58 (0.4)
Fumaric acid	57 (0.4)
Atorvastatin	56 (0.4)
Pregabalin	56 (0.4)
Peginterferon alfa-2b	55 (0.4)
Peginterferon alfa-2a	54 (0.4)
Nicotine	52 (0.4)
Telaprevir	52 (0.4)
Metformin	50 (0.4)
Venlafaxine	49 (0.3)

Sertraline	48 (0.3)
Palbociclib	47 (0.3)
Amoxicillin	46 (0.3)
Fluorouracil	46 (0.3)
Paracetamol	45 (0.3)
Methotrexate	45 (0.3)
Fluoxetine	45 (0.3)
Budesonide	45 (0.3)
Fentanyl	44 (0.3)
Captopril	43 (0.3)
Lamotrigine	42 (0.3)
Pirfenidone	41 (0.3)
Gabapentin	40 (0.3)
Tramadol	40 (0.3)
Dulaglutide	40 (0.3)
Clonazepam	39 (0.3)
Lovastatin	39 (0.3)
Triamcinolone	38 (0.3)
Natalizumab	37 (0.3)
Apremilast	37 (0.3)
Dupilumab	37 (0.3)
Quetiapine	36 (0.3)
Amoxicillin;Clavulanic acid	36 (0.3)
Simvastatin	35 (0.2)
Budesonide;Formoterol	35 (0.2)
Valproic acid	35 (0.2)
Interferon beta-1a	35 (0.2)
Cromoglicic acid	34 (0.2)
Amlodipine	33 (0.2)
Tiotropium	33 (0.2)
Alendronic acid	33 (0.2)
Metamizole	32 (0.2)
Prednisone	31 (0.2)
Cetirizine	31 (0.2)
Fluticasone;Salmeterol	31 (0.2)
Citalopram	31 (0.2)
Enalapril	31 (0.2)
Levetiracetam	31 (0.2)
Cyclophosphamide	31 (0.2)
Lisinopril	30 (0.2)
Rosuvastatin	30 (0.2)
Clindamycin	30 (0.2)
Liraglutide	30 (0.2)
